# Wearable sensing and mobile devices: the future of post-concussion monitoring?

**DOI:** 10.2217/cnc-2016-0025

**Published:** 2017-02-08

**Authors:** William Johnston, Cailbhe Doherty, Fionn Cleirigh Büttner, Brian Caulfield

**Affiliations:** 1 School of Public Health, Physiotherapy & Sports Science, University College Dublin, Ireland; 2 Insight Centre for Data Analytics, University College Dublin, Ireland

**Keywords:** concussion, long-term monitoring, mobile technology, post-concussion syndrome, smartphone, wearable sensor

In the past decade, concussion has received large amounts of attention in public, medical and research circles. While our understanding of the nature and management of concussion has greatly improved, there are still major limitations which need to be addressed surrounding the identification of the injury, determining when an individual is safe to return to normal activity, and what factors may contribute to the development of post-concussion syndrome (PCS).

The current model of concussion management involves a triage evaluation in the acute stage of injury, focusing on the classic signs and symptoms of concussion. Next, the clinician attempts to evaluate key components of cerebral function through clinical symptom evaluation, and traditional assessments of motor and neurocognitive function [1]. The development of the sports concussion assessment tool saw a massive leap forward in the strategies employed in the management of concussion, as it acknowledged the multi-factorial nature of concussion, and provided a standardized means for clinicians to assess the many domains of cerebral function [2]. While these methods have demonstrated some promise in the acute stage, they are not designed for serial monitoring (particularly in instances where PCS develops) [3], and provide us with very little clinically relevant information that can assist clinicians in the return to learn/sport/performance process.

The traditional model of concussion assessment coincides with a graduated return-to-play protocol. This protocol is simply dictated by the length of time since the injury, and symptom resolution with physiological exertion [4]; it does not reflect the athlete's true readiness to return to sport, as determined by a multi-modal objective assessment of the variety of impairments that manifest following concussion or during PCS. Indeed, this methodology is fraught with a number of key limitations: these assessments represent the individual's status at discrete points in time, are focused on quantifying parameters that are subject to a level of hourly and daily variability independent of the concussive injury, and do not acknowledge the heterogeneous and evolving nature of the injury. In addition, while we know that concussion affects short-term physical (such as balance and gait) and cognitive (such as memory and concentration) competencies, the evolving nature of PCS for these competencies is not well understood. There is a dearth of evidence quantifying exactly how an injury such as concussion, with widespread symptomatology, disturbs an individual's capacity for physical activity. Improving the evidence base in these areas is vital considering recent evidence which has suggested that concussion has long term effects on physical competencies, with increased musculoskeletal injury rates being observed for 3/12 months’ post injury [5–7]. Furthermore, concern has been raised as to the long term effects of repeated concussions on cognitive function; however, at this time scientific evidence to support these views is limited [8].

To establish the short- and long-term effects that concussion/PCS may have, clinicians and researchers need to focus on developing ‘multi-modal’ assessment models that elucidate the broad-spectrum of deficits which may inhibit an individual's physical activity capacity. These models can be optimized by adopting a ‘multi-point’ assessment window, to account for the variability and heterogeneity of the recovery process.

Thus, we contend that there is a need to move to a new paradigm in concussion assessment and evaluation – the ‘multi-point–multi-modal’ model. Doing so would incorporate deployment of a battery of assessments that are not only specific to the stage of recovery, but are capable of sufficiently challenging the various domains of physical activity capacity to determine if an individual is ready to progress to the next stage in the return to learn/sport/performance process. Following thorough curricula review and extensive consultations with researchers and practitioners in a variety of healthcare contexts involved in the management of concussion, we have based the ‘multi-point–multi-modal’ framework around an individual's capacity for physical activity. This capacity is underpinned by three competencies:

- Physical (related to physiological and motor performance measures);
- Cognitive (related to concentration, memory and cognitive load); and
- Behavioral (related to daily-living physical activity behaviors).

As we realize the effect that concussion has on these domains, there is a challenge related to how best to measure them. The digital revolution of the 21st century has resulted in an exponential growth in the amount of technology available to the general population. Recent advances in smartphone and wearable sensor technologies have provided researchers and clinicians with the capacity to bring a completely new paradigm to the management of various patient populations [9]. The smartphone can serve as a mobile sensing, data processing, computing and feedback device that is carried by the patient and serves as a vital link between the patient and clinical stakeholders on a 24/7 basis. It brings the laboratory and clinical expertise into the patient's daily life, and affords the clinical team the opportunity to understand how the patient is progressing in the physical, cognitive and behavioral domains proposed in the multi-point–multi-modal assessment model presented in this Editorial.

For instance, in the initial 72-hours post injury, such technology could be utilized to capture detailed measures derived from traditionally used assessments related to an individual's symptoms, behaviors and cognitive/motor function. Likewise, as the individual progresses past this acute stage, such resources may provide a means to deliver standardized time-relevant assessments for each of these domains. For instance, in the physical domain, sensors such as accelerometers, gyroscopes and magnetometers provide an accessible means to obtain micro-level detail surrounding a person's balance during traditional static measures such as the balance error scoring system [10,11]. In addition, the use of such sensor technology during more dynamic assessments such as the Y Balance Test may capture vital information about an individual's balance during more challenging sport specific tasks [12]. The objective quantification of both static and dynamic balance tasks using sensor technology may provide a means to objectively evaluate the function and integration of the motor control subsystems as the individual progresses through the different stages of recovery [13].

In the behavioral domain, biophysical sensors provide the means to acquire longitudinal data regarding an individual's ongoing behaviors during daily life (e.g., sleep, patterns of ambulatory activity and heart rate variability).

These physical competencies and behavioral data could be leveraged to provide macro-level information surrounding a person's true activity levels, and micro-level physiological information relating to autonomic function. When combined with self-reported information such as symptomatology, a contextually rich dataset is derived which may help clinicians and researchers determine if someone is truly recovering, and if not, what factors may be contributing to their development of PCS.

In the cognitive domain, the traditional assessments currently included in the sports concussion assessment tool could be delivered via mobile platforms in the acute stage of concussion, covering the domains of orientation, immediate memory and concentration [2]. Randomization algorithms could be applied through cloud computing to account for the learning effect of such cognitive assessments, providing appropriate variations to the current assessments to ensure accurate results. As the recovery progresses, more advanced evaluation of key neurophysiological domains such as verbal memory, visual memory, visuomotor speed and reaction time, as is commonly assessed in computerized neurophysiological testing [4] could be delivered using mobile platforms.

It is important to highlight that this editorial simply presents a concept: that mobile technology, if leveraged appropriately, could provide both researchers and clinicians with the means to capture large amounts of information across several domains of impairment post-concussion. We contend that concussion assessment and management should be multi-modal and multi-point, thus providing an overarching view of concussion-associated impairments and their recovery. However, it is currently unclear what specific suite of measures may provide us with the most clinically relevant information. Still, smartphone technology has the potential to completely transform the current approach utilized in the management of concussion in several ways. First, it allows clinicians and researchers to objectively measure the influence concussion has across the proposed domains of physical activity competence outside of the laboratory setting. Second, it has the potential to provide valuable information surrounding an individual's rate and pattern of improvement during the recovery process. Third, this technology enables us to capture healthy baseline data that allows us to investigate the ‘healthy’ steady-state across different domains for the purposes of post-injury comparison and understanding the patterns of normal biological variation. Finally, it provides the means to objectively investigate the relationship between different measures and the propensity for delayed recovery and the development of PCS.

Despite all this promise and potential, it would be a mistake to move too quickly toward developing new digitally enabled assessment protocols. The next phase in achieving the goal of a digitally enabled multi-modal–multi-point assessment paradigm for concussion is to engage in a broad-based data gathering program. Only by doing this can we fully ascertain how this new paradigm can realize true value in determining which measurement variables best describe the recovery process, how the interplay between different measurement domains impacts on recovery and how to predict who is at risk of developing PCS. Once this is achieved, we can move toward a roll-out of the digitally enabled assessment batteries that will transform how we monitor and manage concussion. Right now, we encourage researchers and clinicians to engage in interdisciplinary research to take the next step described above.
